# Methanodibenzo[*b*,*f*][1,5]dioxocins as Novel Glutaminase Inhibitor with Anti-Glioblastoma Potential

**DOI:** 10.3390/cancers15041010

**Published:** 2023-02-05

**Authors:** Akshaya Murugesan, Sana Kari, Anita Shrestha, Benedicta Assoah, Konda Mani Saravanan, Monica Murugesan, Ramesh Thiyagarajan, Nuno R. Candeias, Meenakshisundaram Kandhavelu

**Affiliations:** 1 Molecular Signaling Group, Faculty of Medicine and Health Technology, Tampere University and BioMediTech, 33101 Tampere, Finland; 2 Department of Biotechnology, Lady Doak College, Thallakulam, Madurai 625002, India; 3 Faculty of Engineering and Natural Sciences, Tampere University, 33101 Tampere, Finland; 4 Department of Biotechnology, Bharath Institute of Higher Education & Research, Chennai 600073, India; 5 Department of Basic Medical Sciences, College of Medicine, Prince Sattam bin Abdulaziz University, Al-Kharj 11942, Saudi Arabia; 6 LAQV-REQUIMTE, Department of Chemistry, University of Aveiro, 3810-193 Aveiro, Portugal

**Keywords:** glutaminase inhibitors, new dioxocin derivatives, synthesis, characterization, signaling, interaction, modeling

## Abstract

**Simple Summary:**

The glutaminolysis pathway is recognized as one of the hallmarks of glioblastoma associated with tumor cell maintenance, survival, and aggressiveness. Targeting glutaminolysis emerged as a promising strategy for tumor treatments. Still, the development of glutaminase inhibitors is limited, which demands the identification of novel inhibitors for disrupting glioblastoma metabolism and its progression. Here, we report a novel library of dioxocin derivatives as glutaminase inhibitors and their pharmacological intervention for treating glioblastoma.

**Abstract:**

Glutamine metabolism is an important hallmark of several cancers with demonstrated antitumor activity in glioblastoma cancer cells (GBM). GBM cells regulate glutamine and use it as a major energy source for their proliferation through the glutaminolysis process. Enzymes, such as glutaminase in glutaminolysis, can be targeted by small-molecule inhibitors, thus exhibiting promising anticancer properties. The resistance to glutaminolysis demands the development of new therapeutic molecules to overcome drug resistance. Herein, we have reported a novel library of constrained methanodibenzo[*b*,*f*][1,5]dioxocin derivatives as glutaminase (GLS) inhibitors and their anti-GBM potential. The library consisting of seven molecules was obtained through self-condensation of 2′-hydroxyacetophenones, out of which three molecules, namely compounds 3, 5, and 6, were identified with higher binding energy values ranging between −10.2 and −9.8 kcal/mol with GLS (PDB ID; 4O7D). Pharmacological validation of these compounds also showed a higher growth inhibition effect in GBM cells than the standard drug temozolomide (TMZ). The most promising compound, 6, obeyed Lipinski’s rule of five and was identified to interact with key residues Arg^307^, Asp^326^, Lys^328^, Lys^399^, and Glu^403^ of GLS. This compound exhibited the best cytotoxic effect with IC_50_ values of 63 µM and 83 µM in LN229 and SNB19 cells, respectively. The potential activation of GLS by the best-constrained dibenzo[*b*,*f*][1,5]dioxocin in the tested series increased apoptosis via reactive oxygen species production in both GBM cells, and exhibited anti-migratory and anti-proliferative properties over time in both cell lines. Our results highlight the activation mechanism of a dibenzo[*b*,*f*][1,5]dioxocin from the structural basis and demonstrate that inhibition of glutaminolysis may facilitate the pharmacological intervention for GBM treatment.

## 1. Introduction

Glioblastoma multiforme (GBM), specifically grade IV astrocytoma, is one of the fast-growing and aggressive brain tumors, which starts from either the brain or the spinal cord. It shares common characteristics with other tumors, such as uncontrolled proliferation, evasion of apoptosis, invasiveness, avoidance of immune surveillance, resistance to chemotherapy, and angiogenesis [1,2]. Glutamine is an important energy substrate and carbon source for cancer cells, with glutamine “addiction” emerging as a hallmark of many cancers, including GBM. Glutamine is catabolized by glutaminase 1 (GLS1), carbamoyl-phosphate synthetase 2-aspartate transcarbamylase-dihydroorotase (CAD), or glutamine fructose-6-phosphate amidotransferase (GFAT). Glutaminase (GLS) is amenable for glutaminolysis, which is a process harnessed by cancer cells to feed their accelerated growth and proliferation in many malignant tumors. α-Ketoglutarate (α-KG) is an intermediate of the tricarboxylic acid (TCA) cycle [3]. Most primary grade II and III infiltrating gliomas and secondary glioblastomas (grade IV) exhibit mutations at the isocitrate dehydrogenase genes (IDH) that produce 2-hydroxyglutarate (2-HG) rather than α-KG. α-KG provides citrate for acetyl-CoA synthesis, an essential substrate of fatty acid synthesis.

**Scheme 1:**
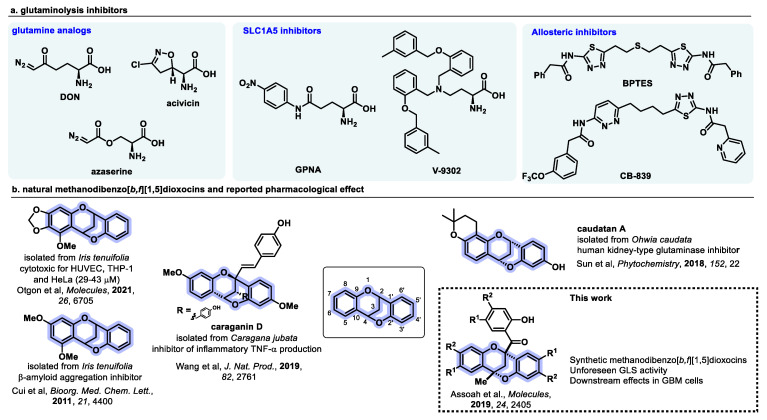
(**a**) Representative examples of reported glutaminolysis inhibitors: DON = 6-diazo-5-oxo-L-norleucine, GPNA = L-γ-glutamyl-*p*-nitroanilide, BPTES = Bis-2-(5-phenylacetamido-1,3,4-thiadiazol-2-yl)ethyl sulfide. (**b**) Previously reported biologically active natural methanodibenzo[*b*,*f*][1,5]dioxocins.

Several drugs have been developed to inhibit different parts of the glutaminolysis pathway (Scheme 1a). The first type is exemplified by glutamine analogs, such as 6-diazo-5-oxo-L-norleucine (DON), acivicin, and azaserine [4,5,6,7]. The second group, represented by ASCT2 (SLC1A5) inhibitors, includes GPNA and V-9302, while the most extensively studied inhibitor of GLS is based on the BPTES and CB-839 molecular scaffolds [8]. The GLS1 gene is overexpressed in many tumor cell lines and primary tumors, while the GLS2 gene is not widely expressed in tumors. Efficient inhibition or genetic silencing of GLS1 in different tumor models has validated GLS1 as a therapeutic target, making it a better target for glutaminolysis inhibition than GLS2 [9]. The glutamine pathway is emerging as an important counterpart for cancer prognosis and a target for new treatments [10]. 

Despite the developments in GLS inhibitors, notable setbacks in the development of pro-drugs greatly driven by the lack of selectivity and poor bioavailability causes some cancer cells to show resistance to glutaminase inhibitors. Thus, the search for potent and selective GLS inhibitors remains an open issue. Flavonoids are ubiquitously found in plants, and their biological properties have been widely explored. Although they are mostly studied for their radical scavenging and antioxidant ability, other properties such as: anti-inflammatory, anticancer, cardioprotective, antimicrobial, and antiviral, have been disclosed [11,12,13]. C4-cycloflavans containing the methanodibenzo[*b*,*f*][1,5]dioxocin skeleton found in biologically active natural products and pharmaceuticals (Scheme 1b) are a subset of such privileged class of flavonoids. Among other interesting properties, methanodibenzo[*b*,*f*][1,5]dioxocins have been shown to inhibit activity against β-amyloid aggregation and bacterial growth inhibition [14]. When considering the modification of the methylene bridge, the pharmacological properties of these types of compounds are expanded to also include the anti-inflammatory nitrous oxide formation inhibitor caraganin D [15] and the human kidney-type glutaminase [16] inhibitor caudatan A. 

Despite the interesting biological properties of methanodibenzo[*b*,*f*][1,5]dioxocin derivatives isolated from natural sources, this motif has received little attention in drug design, likely because of the limited number of methods for its preparation. The highly complex scaffold can be obtained from the dimerization of salicylaldehydes [17,18], and most recently, Tan and co-workers have ingeniously explored an olefin isomerization/hemi-acetalization/dehydration/[3+3]-type cycloaddition cascade sequence to introduce diversification, mostly on the aromatic substituents [19,20,21]. Other cascade processes from in situ-generated alkynyl *o*-quinone methide and phenols using silver triflate [19] or camphorsulfonic acid [22] as catalysts were demonstrated to provide such [1,5]dioxocins in moderate to excellent yields. We have recently explored [23] the preparation of disubstituted methanodibenzo[*b*,*f*][1,5]dioxocins through a pyrrolidine-catalyzed self-condensation of 2′-hydroxyacetophenones, which afford some diversification at position 2 and aromatic substituents on the methanodibenzo[*b*,*f*][1,5]dioxocin structural motif. Aware of the limited availability of such intricate compounds, and motivated by the structural resemblance between our small library of compounds with the human kidney-type glutaminase inhibitor caudatan A, we set out to study the inhibitory properties of the previously synthesized methanodibenzo[*b*,*f*][1,5]dioxocins.

In the present study, we investigated the mechanism of action of dioxocins in glutaminolysis pathway inhibition, their potential anticancer effect in GBM cells (LN229 and SNB19), and further characterized their mode of interaction with GLS. The current study intends to correlate GLS inhibition and the possible downstream effects in GBM cells. The race to develop new therapeutics in this field is discussed to provide a reference for developing a novel glutaminase modulator for the treatment of cancers.

## 2. Methodology

### 2.1. Synthesis of Novel Dioxocin Derivatives

The preparation of methanodibenzo[*b*,*f*][1,5]dioxocins was previously described by Assoah et al. [23]. The round-bottom flask (10 mL), equipped with a condenser, was loaded with the corresponding 2′-hydroxyacetophenone (6.22 mmol) and heated in hexane (5 mL) until complete dissolution. Upon dissolution, pyrrolidine (2.08 mmol, 0.33 equiv) and molecular sieves (3 Å beads, 362 mg) were added, and the mixture was stirred under argon, followed by a 24–48 h reflux (80 °C). The mixture was cooled to room temperature followed by partitioning between ethyl acetate and saturated NH_4_Cl (15 mL). The aqueous layer was extracted with ethyl acetate (3 × 20 mL) and the combined organic layers were dried over MgSO_4_ and filtered out, followed by solvent removal under reduced pressure. The obtained residue was purified by flash column chromatography on silica (Hexane/EtOAc 98:2) to yield the desired product.

### 2.2. Computational Assessment of Ligand–Glutaminase Interaction

Auto-dock Tools (ADT) was used to perform molecular docking of glutaminase, GLS (PDB ID; 4O7D, crystal structure of glutaminase), and dioxocin derivatives (**1–7**). The glutaminase molecule was modified by the incorporation of polar hydrogens, Kollman charges, and AD4-type atoms, while Gasteiger charges were introduced into the ligands and the greatest number of active torsions was preserved. The grid map was made with the help of AutoGrid. All docking processes had a uniform grid spacing of 0.375. Active sites were specified in accordance with the location of the small molecule in the crystal complex structure. Glutaminase was kept stiff throughout the docking using the Lamarckian Genetic Algorithm (LGA). The population size was set to 150, with individuals seeded at random, and the maximum number of energy assessments was set to 500,000. The rest of the docking settings were left at their defaults. After generating ten alternative postures for each ligand, the scoring procedures in AutoDock 4.2 were utilized to determine which ones had the lowest docked energy, and they were ranked accordingly.

### 2.3. Absorption, Distribution, Metabolism, Elimination, and Toxicity (ADMET) Prediction

The in silico ADMET study focused on the human body’s pharmacokinetic properties of dioxocins **1–7**. In this study, SwissADME (http://www.swissadme.ch/, accessed on 1 October 2022) and pkCSM (https://biosig.lab.uq.edu.au/pkcsm/prediction, accessed on 1 October 2022) online software tools were used to predict ADMET properties and toxicity, respectively. SwissADME, developed by the Swiss Institute of Bioinformatics, is a hybrid web server that predicts and analyzes the ADMET properties of numerous compounds. This software reveals the physicochemical properties such as lipophilicity, water solubility, drug likeness, etc.,[24], while pkCSM tools revealed the toxicity of the structural ligand. Specifically, the following parameters were calculated with their standard ranges: in absorption, the colon cancer cell line permeability (CaCO_2_) and intestinal absorption; in distribution of drugs, the blood–brain barrier (BBB) and CNS permeability; in metabolism, CYP2D6, CYP3A4, CYP1A2, CYP2C19, CYP2C9, CYP2D6, and CYP3A4 models; in excretion, the total clearance and renal OCT2 substrate model, and in toxicity, AMES toxicity and hepatotoxicity models were checked.

### 2.4. Cell Culture Materials

Dulbecco’s modified eagle medium (DMEM) (Sigma-Aldrich, St.Louis, MO), fetal bovine serum (FBS) (Sigma-Aldrich, St. Louis, MO,#F7524), L-glutamine (Sigma-Aldrich, St. Louis, MO #G7513), penicillin and streptomycin (Sigma-Aldrich, St. Louis, MO, P4333), amphotericin B (Sigma-Aldrich, St. Louis, MO, #A9528), Trypan blue solution (Sigma-Aldrich, St. Louis, MO, #T8154), propidium iodide (Molecular Probes by Life Technologies, Eugene, OR, #P3566), trypsin-EDTA solution (Sigma-Aldrich, St. Louis, MO, #59418C), Dead Cell Apoptosis Kit with Annexin V FITC and PI (Thermo Fisher Scientific), reactive oxygen species detection reagent (Molecular Probes by Life Technologies, Eugene, OR, #C13293), dimethyl sulphoxide (DMSO) Hybri-Max (Sigma-Aldrich, St. Louis, MO, #D2650), and temozolomide (TMZ) (Sigma-Aldrich, USA) were used.

### 2.5. Cell Culture and Drug Preparation

The human glioblastoma cell lines SNB19 and LN229 (ATCC^®®^ CRL2611™/CRL-2611™, kindly gifted by Dr. Kirsi Rautajoki, Faculty of Medicine and Health Technology, Tampere), and non-cancerous cells, mouse embryonic fibroblast cell line (MEF cell line, kindly gifted by Prof. Pasi Kallio, Faculty of Medicine and Health Technology, Tampere), were cultured in DMEM, as previously reported [2]. The LN229 and SNB19 cell lines were derived from right and left frontal parieto-occipital GBM patients, respectively. For testing the compounds, synthesized methanodibenzo[*b*,*f*][1,5]dioxocins **1–7** and TMZ, a chemotherapeutic agent for glioblastoma treatment, were dissolved in DMSO to obtain a final concentration of 100 mM, from which intermediate dilutions were prepared. For the dose-dependent cytotoxicity assay, the final concentrations of 150 μM, 100 μM, 75 μM, 50 μM, 25 μM, and 10 μM were prepared in the same culture medium. 

### 2.6. Measuring Plasma Membrane Integrity with Trypan Blue

**Figure 1:**
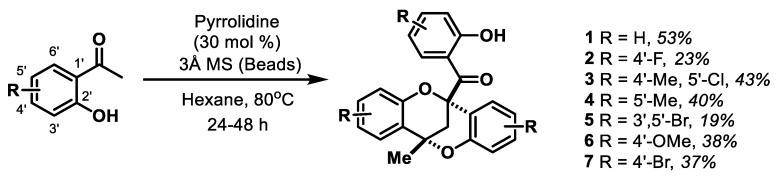
Synthesis of methanodibenzo[*b*,*f*][1,5]dioxocins **1–7**.

The cytotoxic effects of dioxocins **1–7**, reported in Figure 1, were studied on glioblastoma cell lines, SNB19 and LN229. For this, cells were seeded on 12-well plates with a density of 1 × 10^5^ cell/well. After 48 h, the cells were treated with a 100 μM concentration of the compounds of interest. TMZ and DMSO (0.1%) were used as positive (PC) and negative controls, respectively. The cytotoxicity effect of 100 µM of the top lead compound was also tested against the growth of normal cells, MEF. The Trypan blue assay allows to measure the plasma membrane integrity, which is a widely used method for determining cell viability [24,25,26]. To quantify live and dead cell populations, a Bürkerhemo-cytometer (Heinz Herenz, Hamburg, and Germany) was used. Biological and technical repeats were used to obtain the statistically significant results.

### 2.7. In Vitro Measurement of Dose-Dependent Cytotoxicity

The cytotoxic effects of the best dioxocins were studied on both LN229 and SNB19 cells. These cell lines were seeded in a 6-well plate with an initial density of 4 × 10^5^ cells and treated with varying concentrations (10 μM, 25 μM, 50 μM, 75 μM, 100 μM, and 150 μM) of the best derivative after the cells reached 70% confluency. The cells were harvested after 48 h and the DMSO was used as the negative control. The total numbers of live and dead cells were counted using the plasma membrane integrity assay, as described above. The compounds’ half-maximum inhibitory concentration (IC_50_) was evaluated with the dose–response curve plotted using GraphPad software (version 6). The percentage of cell growth inhibition was calculated using the formula below:

$$Cell\;Growth\;Inhibition\;\;\left(\%\right)=\frac{Mean\;No.\;of\;DMSO\;treated\;cells-Mean\;No.\;of\;dioxocin\;treated\;cells\;\;}{Mean\;No.of\;DMSO\;treated\;cells}\times 100$$

### 2.8. Reactive Oxygen Species Assay (ROS)

The LN229 and SNB19 cells were seeded in 6-well plates with 4 ×10^5^ cells per well and incubated overnight. The cells were then treated with DMSO, hydrogen peroxide (H_2_O_2_), and the IC_50_ concentration of the compound for 5 h. After that, the cells were collected through centrifugation at a rate of 3000 rpm for 10 min and then transferred to a 96-well plate. The concentration of 2 μM of 2′,7′-dichlorodihydrofluoresceindiacetate (H_2_DCFDA) was then mixed with the cells and incubated for about 20 min. They were then subjected to a pre-warmed medium and again incubated for 20 min after they had been washed with 1X PBS. The fluorescence intensity of the cells was then measured using a plate reader with the excitation and emission wavelengths of 485 nm and 538 nm, respectively. Hydrogen peroxide (H_2_O_2_, 200 μM, Sigma-Aldrich, and St. Louis, MO) was used as the positive control. The fold increase in ROS production was calculated using the following formula [27]:

Fold increase ═ F_rest_ − F_blank_/F_control_ − F_blank_

### 2.9. Apoptosis Assay

To quantify apoptosis in the lead-treated cells, the Dead Cell Apoptosis Kit with Annexin V-FITC and PI was used. The assay was performed as previously reported [28]. Treated cells were incubated for 48 h. Cells were harvested by accutase, followed by centrifugation at 1800 rpm for 5 min before being washed in ice-cold PBS. The cell pellets were resuspended in ice-cold 1X annexin-binding buffer. Annexin V FITC and PI working solutions were added to the cell suspension, as suggested by the manufacturer’s protocol. The cells were then incubated in the dark at room temperature for 15 min prior to the fluorescence measurement. An EVOS imaging system (ThermoFisher Scientific) with 20X objective magnification was used to observe the apoptotic cells, and images were taken for the quantification of apoptotic and non-apoptotic cell percentages [24]. At least 300 cells were observed to reach statistically significant data from different fields under a microscope.

### 2.10. Time-Dependent Effect of Inhibition

To analyze the effect of the lead compound over time, the LN229 and SNB19 were treated with the IC_50_ concentration, and cell death was measured at 24 h, 48 h, and 72 h. In detail, both cell lines were plated in a 6-well plate with a density of 4 × 10^5^ cells. Cultures were incubated overnight before they were treated with the IC_50_ concentration of the lead derivative. The treated cells were collected by centrifugation at a rate of 3000 rpm for 10 min after 24 h, 48 h, and 72 h, respectively. To measure the percentage of live and dead cells, the Trypan blue assay was performed as described above. The live and dead cell counts were then calculated using the Countess II FL Automated Cell Counter (ThermoFisher Scientific, Waltham, MA, USA). The inhibitor percentage was computed using the same equation as described for the in vitro measurement of dose-dependent cytotoxicity.

### 2.11. Cell Migration Assay

To study the effect of the lead compound on cell migration, an initial density of 1 × 10^5^ cells/well was plated on 12-well plates. The scratch assay was performed as previously described [2] and the scratched area was cleaned by washing the cells with 1 mL of phosphate-buffered saline (PBS). Then, 1 mL of culture medium was supplemented with 2% FBS and an IC_50_ concentration of the lead compound and the DMSO control. The cells cultured with medium containing 2% FBS alone were used as a control. The scratched area was visualized by a phase contrast microscope every 2 h for a period of 8 h. To track the cell migration, the image acquisition was performed by using the EVOS imaging system (ThermoFisher Scientific, Waltham, MA, USA) with 10X objective magnification. 

### 2.12. Statistical Analysis

All the biological experiments were performed with three biological and technical repeats. The data were presented as the mean ± standard error of mean. Statistical differences between groups were tested by one-way analysis of variance using Dunnett’s multiple comparison test (GraphPad Prism ver. 7.04, San Diego, CA, USA). *p* < 0.05 was considered to indicate statistically significant data.

## 3. Result

### 3.1. Dioxocins Interact with Human Glutaminase

Dioxocin derivatives **1–7** were prepared according to Scheme reported in Figure 1, as reported elsewhere [23]. The pyrrolidine-catalyzed condensation of 2’-hydroxyacetophenone and aryl-substituted congeners was performed in hexane at 80 °C in the presence of 3 Å molecular sieves for 24–48 h. Notwithstanding the moderate isolated yields (19–53%), the desired compounds were obtained as pure single diastereomers after chromatographic purification, as determined by ^1^H NMR, and the structures were further confirmed by ^13^C NMR characterization and HRMS.

To obtain a deeper insight into the interaction of all novel derivatives, a docking study was carried out against human glutaminase protein, GLS. Autodock Vina docking was performed to rank the best compounds binding with glutaminase. All compounds docked in Autodock Vina showed scores lower than −8.0 kcal/mol, and these results are shown in Table 1. The results clearly reveal that dioxocins bind relatively well with glutaminase. The binding energy of all seven derivatives has been provided in Table 1. From our results, we observed that **3** (MW = 517.83 g/mol), **5** (MW = 845.8 g/mol), and **6** (MW = 462.5 g/mol) potentially interact with human glutaminase, with docking scores of −10.248, −10.021, and −9.820, respectively (Table 1). Thus, the top three protein–ligand complexes (**3**, **5,** and **6**) were selected for further investigation based on the autodocking score. 

**Figure 2:**
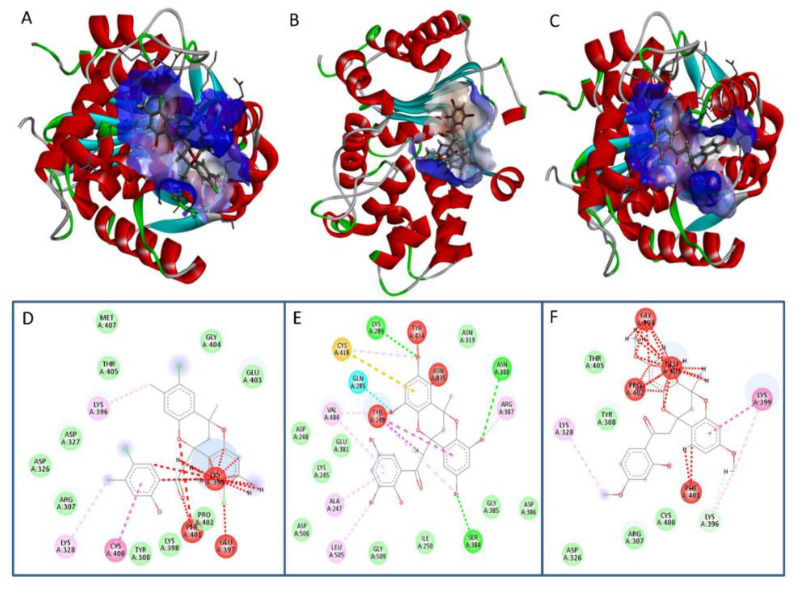
Three-dimensional representation of top docking poses of: (**A**) glutaminase-**3**, (**B**) glutaminase-**5**, and (**C**) glutaminase-**6** complexes. The two-dimensional chemical interaction diagrams of: (**D**) glutaminase-**3**, (**E**) glutaminase-**5,** and (**F**) glutaminase-**6** complexes.

The three- (Figure 2A–C) and two-dimensional (Figure 2D–F) models of the top three compounds bound in the glutaminase pocket were further analyzed. The ligand strongly bound to the target protein by forming stabilizing interactions. The two-dimensional representation (Figure 2E) of hexabrominated derivative **5** shows a greater number of interactions (21 contacts with amino acid residues) with the glutaminase amino acid residues than other compounds (**3**: 16 interactions (Figure 2D) and **6**: 12 interactions (Figure 2F)). As seen in the image, the formation of protein–ligand complexes was dominated by van der Waals interactions. Traditional hydrogen bonds are shown as dark green, whereas van der Waals bonds are shown in a lighter shade of green. Upon complex formation, the glutaminase residue tyrosine at various locations forms van der Waals interactions with the ligand. The major interactions between the protein and the ligands are van der Waals interactions, which only occur when neighboring atoms are in close contact with one another. Glutaminase residues Lys, Cys, and Tyr come into close proximity to hydrophobic groups in the top three compounds, **3** (Figure 2D), **5** (Figure 2E), and **6** (Figure 2F). It was noted that the charged residues Glu and Arg interact with all three compounds, which strongly stabilizes the interactions between the glutaminase and the compounds.

### 3.2. Drug-Likeness Properties of Dioxocin Analogs

From the ADMET analysis of dioxocin analogs, we predicted that **1** and **4–6** showed higher human intestinal absorption (HIA), indicating that these orally administered compounds can be effectively absorbed (Table 2). Water solubility estimation revealed that **1**, **2,** and **6** were moderately soluble, while other compounds were poorly soluble, except **5** which was insoluble. The CaCO_2_ cell assay is one of the most important assays which predicts intestinal drug permeability and absorption. The in silico CaCO_2_ permeability models allow to predict problematic drugs. Compounds **1** and **4–6** were estimated to be CaCO_2_-permeable, which indicates their absorption ability. Compound **1** was estimated as efficient in crossing the blood–brain barrier region, thus indicating its potential use as a neurodegenerative drug. Dioxocins **1**, **5,** and **7** were found to be CYPA12 inhibitors. The results also suggest that compounds **1–7** showed no toxic effect in the AMES test, while **1, 2, 4,** and **7** may possess a hepatotoxic effect.

Drug likeness evaluates the bioavailability of the drug, which assesses a molecule as an oral drug. The tested set of compounds reveals only two violations in Lipinski’s rule for compounds **3**, **5,** and **7,** while other compounds showed none or one violation, indicating that all these compounds act in accordance with the rule of five [29]. From the above predictions, it is clearly evident that **1** and **6** possess all the ADMET properties to act as drug-like compounds.

### 3.3. Dioxocin 6 Effectively Reduces Cell Viability of GBM Cells

**Figure 3:**
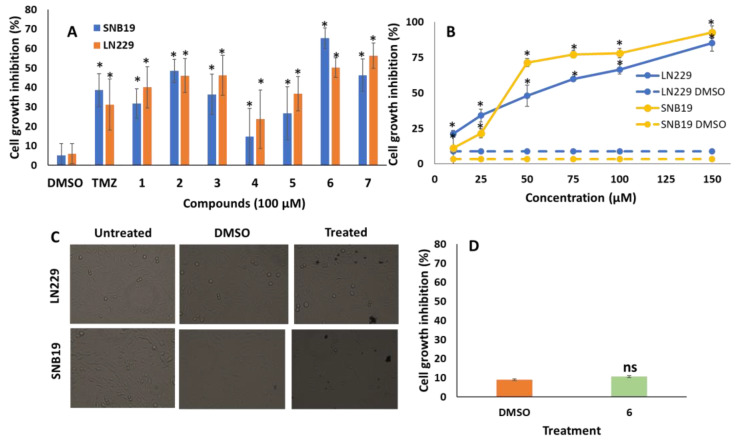
(**A**) Effect of compounds **1–7** against the growth of GBM cells, LN229 and SNB19. (**B**) Effect of compound **6** in LN229 and SNB19 cells. Percentage of cell viability for LN229 and SNB19 cell lines upon treatment with compound **6** at different concentrations, 10 μM, 25 μM, 50 μM, 75 μM, 100 μM, and 150 μM. Datapoints and error bars represent mean ± S.E.M (n = 6). (**C**) Phase contrast images of GBM cells in untreated, DMSO-treated, and IC_50_ concentration of compound **6**. (**D**) Percentage of cell growth inhibition in the MEF cell line treated with compound **6** at a 100 μM concentration. Datapoints and error bars represent mean ± S.E.M (n = 6). * *p* < 0.05 relative to the respective DMSO control. ns, statistically non-significant.

The experimental validation was performed by determining the cell growth inhibition of compounds **1–7** against GBM cells, specifically, SNB19 and LN229 cell lines. Delightfully, compounds **1–3**, **6,** and **7** showed higher inhibition at a 100 µM concentration (Figure 3A). At this concentration, compounds **2**, **3**, and **7** exhibited more than 50% cell growth inhibition. On the other hand, compounds **4** and **5** were less effective against GBM cells and the inhibition remained less, with only 10–40% growth inhibition, although **5** remained slightly more effective than the positive control. Interestingly, compound **6** proved to be the most promising compound, with an inhibition of 65% in SNB19 and 50% in LN229 cell growth, which is more effective than TMZ (positive control). Considering the non-violation of Lipinski’s rule of five by compound **6** and the reliable inhibitory activity tested, this compound was selected for further analysis. Notably, compound **6** interacted with GLS residues of Arg^307^, Asp^326^, Lys^328^, Lys^399^, and Glu^403^ (Figure 2F).

### 3.4. Dose-Dependent Effect of Compound 6 in GBM Cells

To determine the half-maximal inhibitory concentration (IC_50_), LN229 and SNB19 cells were subjected to treatments with solutions of compound **6** at concentrations of 10 μM, 25 μM, 75 μM, 100 μM, and 150 μM, as described in the Methods Section. The calculated IC_50_ values of the tested compound are shown in Figure 3B, with a value of 63.12 µM for SNB19 and 83.54 µM for LN229 cells. In detail, the lowest concentrations of inhibition were 21% and 11% at 10 µM and ~50% and 70% at 50 µM for SNB19 and LN229 cells, respectively. The compound showed the highest inhibition of 85% and 93% in 150 µM for LN229 and SNB19 compared with DMSO (Figure 3B). Further, microscopic observation of cells treated with the IC_50_ concentration showed higher cell growth inhibition in both LN229 and SNB19 cells compared with untreated and DMSO-treated cells (Figure 3C). Upon 100 μM treatment, compound **6** showed ~11% growth inhibition against the growth of non-tumorous cells, MEF (Figure 3D). These results suggest that compound **6** induced less of a cytotoxic effect in non-cancerous cells than the GBM cells. Based on these results, compound **6** was selected for further investigation on the mechanism of action in GBM cells.

### 3.5. Interaction of Compound 6 with Glutaminase Elevates Intracellular ROS and Apoptosis in GBM Cells

**Figure 4:**
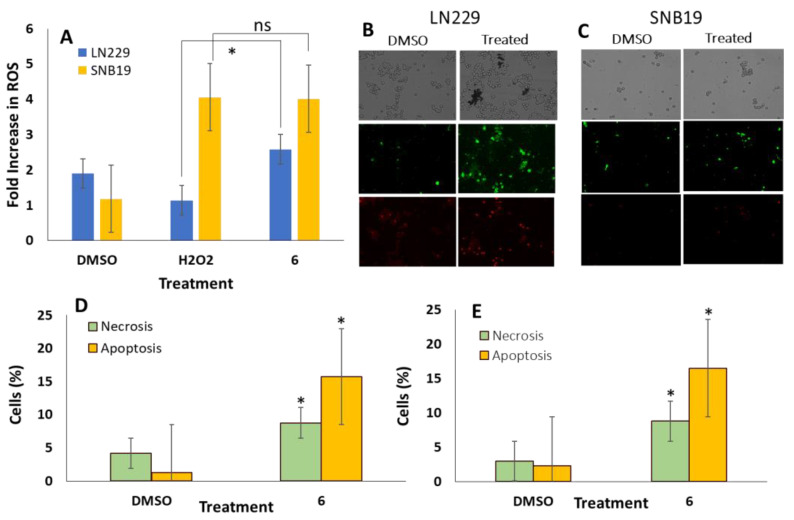
Validation of compound **6**-induced ROS changes in GBM cells. (**A**) Effect of compound **6** in intracellular ROS production in LN229 and SNB19 cells. Cells were treated with the IC_50_ concentration for 5 h and intracellular ROS was measured from the fluorescence intensity of H_2_DCFDA. DMSO and H_2_O_2_ were used as negative and positive controls, respectively. (**B**,**C**) Microscopic images of the cells stained with Annexin V-FITC and PI in (**B**) LN229 and (**C**) SNB19 cells. Image panels: DMSO- and compound **6**-treated cells. (**D**,**E**) Percentage of apoptotic and necrotic cells in DMSO- and compound **6**-treated conditions for (**D**) LN229 and (**E**) SNB19 cells. Datapoints and error bars represent mean ± S.E.M (n = 6). * *p* < 0.05 relative to the respective control. ns, statistically non-significant.

To determine the glutaminase inhibitory effect, the levels of reactive oxygen species (ROS) in LN229 and SNB19 cells were measured upon treatment with compound **6** and H_2_O_2_ [27]. Interestingly, an increase of ROS species was observed in both GBM cells upon treatment with compound **6** (Figure 4A). The LN229 and SNB19 cells treated with compound **6** increased their ROS production by ~2.5-fold compared with DMSO (Figure 4A). Additionally, the ROS level in LN229 cells treated with compound **6** was higher than those with hydrogen peroxide. Similarly, compound **6** produced a ~4.0-fold increase in SNB19 cells when compared with the untreated and H_2_O_2_-treated cells. Overall, the data suggest that the significant increase in ROS production in GBM cells was induced by compound **6**. The elevated level of ROS validates compound **6** as a glutaminase inhibitor, which could be interlinked with the observed GBM cell death.

The effect of compound **6** in inducing apoptosis was measured using Annexin V/PI double staining in both LN229 (Figure 4B) and SNB19 cells (Figure 4C). The microscopic imaging shows a higher percentage of Annexin V/PI-positive cells treated with compound **6** compared with DMSO. 

These results revealed that compound **6** triggers apoptosis (Annexin V/PI-positive cells) and necrosis (PI-positive cells) in LN229 (Figure 4D) and SNB19 cells (Figure 4E). In detail, the apoptosis percentage of compound **6**-treated LN229 cells showed 15.70% of apoptotic cells, while the control (DMSO) exhibited 1.25%. Additionally, the necrotic cells after treatment with compound **6** were observed to be 8.77%, greater than DMSO. In SNB19 cells, 16.48% and 2.33% of apoptotic cells were observed in compound **6**-treated and DMSO-treated cells, respectively. The necrotic cells showed 8.79%, greater than DMSO. These data suggest that compound **6** induced cell death through apoptosis, where elevated ROS might be interlinked in the downstream signaling of glutaminase in GBM cells. 

### 3.6. Compound 6 Inhibits GBM Cell Migration and Proliferation

**Figure 5:**
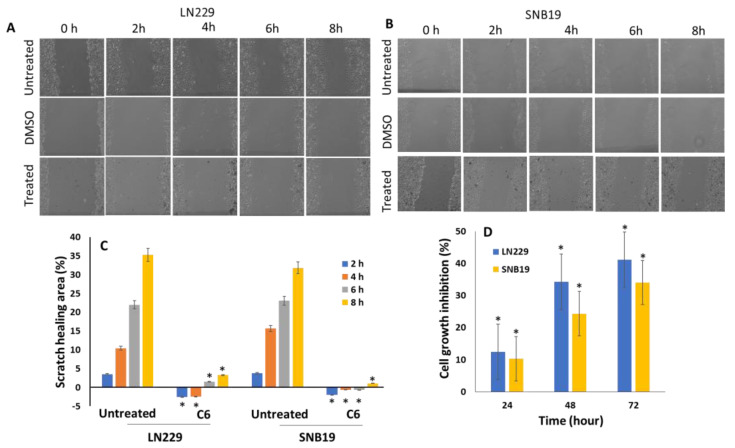
Compound **6** inhibited GBM cell migration and proliferation. Scratch assay was performed in treated GBM cells in an appropriate medium with 2% FBS. Imaging of scratch area for every 2 h up to 8 h. Image shows (**A**) LN229 and (**B**) SNB19 untreated, DMSO-, and compound **6**-treated cells of the scratch assay tracked over time. All images were viewed under a light microscope with a 4X objective (n = 6). (**C**) Quantification of distance migrated after scratching in LN229 and in SNB19 cells. * *p* < 0.05, treated vs. untreated. (**D**) Percentage of GBM cells treated with the IC_50_ concentration for 24 h, 48 h, and 72 h. Datapoints and error bars represent mean ± S.E.M (n = 6). * *p* < 0.05, treated vs. DMSO control.

To determine the effect of **6** on the migration of LN229 (Figure 5A) and SNB19 (Figure 5B) cells, the cell lines were cultured in an appropriate medium with 2% FBS. The images showed the appearance of a disturbed or detached monolayer when treated with compound **6**, whereas the 2D monolayer was not influenced in untreated control cells. Additionlly, a number of adherent cells detached immediately after the treatment with compound **6**. These results suggest that compound **6** inhibits the migration and invasion of LN229 (Figure 5A,C) and SNB19 cells over time (Figure 5B,C). In detail, the lower concentration of FBS validates that the observed migratory response is not due to proliferation. A scratch healing area was also measured every 2 h for a period of 8 h. It is shown that the ability of the cells to migrate to the scratched space significantly decreased when compared to the untreated cells. The analysis showed that migration of cells was inhibited to about ~1% at 2 h post-scratch and 23% at 8 h in SNB19 cells, while LN229 cells showed 2% inhibition at 2 h and 21% at 8 h (Figure 5C). The observed effect of compound **6** was normalized against the DMSO control and compared to untreated cells.

To determine whether compound **6** can inhibit GBM cell proliferation over time, the viability of LN229 and SNB19 cells was measured 24 h, 48 h, and 72 h after treatment, in the presence of 5% FBS (Figure 5D). The lesser % of inhibition of about 12% and 11% at 24 h, and an average growth inhibition of about 35% and 25%, were observed at 48 h in LN229 and SNB19 cells, respectively. The highest % of inhibition was found to be about 41% in LN229 and 35% in SNB19 cells at the time interval of 72 h, where the invaded areas increased steadily over time. Overall, these results indicate that compound **6** showed potential inhibition of GBM’s cell growth in a time-dependent manner.

## 4. Discussion

Glutaminase plays a crucial role in the metabolism of numerous cancers, including GBM, through the glutaminolysis pathway. Glutaminolysis is one of the major altered metabolic pathways involved in tumor growth [30,31,32] and a high glutamine concentration has been associated with cell transformation. The glutamine metabolism pathway is emerging as an important counterpart in cancer prognosis and a new target for treatments. The mammalian target of rapamycin (mTOR) signaling pathway plays a critical role in tumor metabolism by regulating cell proliferation, autophagy, and apoptosis. Hence, the mTOR signaling pathway is also targeted in anticancer research, where a combination of mTOR inhibitors with drugs have proven to be effective in cancer therapy [33]. Additionally, studies suggest that glutaminase and mTOR inhibition causes GBM cell death and tumor inhibition in a xenograft model, resulting in an increased intracellular glutamate level and upregulation of glutaminase in GBM U87MG, and to a greater extent in U87/EGFRvIII cells [34]. Here, we explored the pharmacokinetic interactions of novel dioxocin derivatives with glutaminase and their anti-GBM potential. Among several dioxocin derivatives, the one derived from the condensation of 2’-hydroxy acetophenones decorated with a 4′-methoxy or a 4′-bromo substituent (i.e., dioxocins **6** and **7**, respectively) showed a better inhibition effect in LN229 and SNB19 cells. The presence of other halides at different positions of the aromatic rings or methyl substituents was not beneficial to confer the better inhibitory properties. As our findings suggested that the docking of compounds **3**, **5**, or **6** with glutaminase strongly interacted with the receptor, methanodibenzo[*b*,*f*][1,5]dioxocins can serve as potent glutaminase inhibitor lead compounds. However, considering the distinct interactions observed for these three compounds, a detailed study on the molecular diversity is further required to describe the structure–activity relationships. Nevertheless, the cell growth inhibition effect and bioavailability prediction described compound **6** as a potential lead among the other tested compounds. 

Compound **6** showed a significantly high cytotoxic effect in LN229 cells, with an IC_50_ of ~83.52 µM, and the SNB19 cell line, with IC_50_ of ~63.12 µM, which is in a close range to other synthetic glutamine compounds (IC_50_ of ~85–95 µM for indole derivatives [35] and natural caudatan A, IC_50_ of ~37 µM [16]). The pharmacokinetic prediction revealed that compound **6** has good human intestinal absorption (HIA). The HIA plays an important role in transporting drugs into the human body and is readily absorbed by the intestine [36]. Dioxocins **3** and **5** also showed higher HIA, indicating that these orally administered compounds can be effectively absorbed.

Reactive oxygen species, such as hydrogen peroxide, hydroxyl radicals, and superoxide anions, produced in living cells play a significant role in many cellular functions [37], including genetic mutation and genetic instability, resulting in cellular damage [38,39,40,41]. Increased ROS levels result in oncogene stimulation and enhanced metabolism in cancer cells [37]. Our findings revealed a significant increase in the ROS level exerted by compound **6,** and this might be a plausible cause for the apoptotic pathway induction through glutaminolysis inhibition. Although compound **6** is considered a promising lead, further pharmacological and biological studies are required to fully disclose the mechanism of action of glutaminase inhibitor **6** in glioblastoma cells. Though allosteric inhibitors such as BPTES and CB-839 showed significant inhibition in cancer cell growth targeting glutaminolysis, their low bioavailability restricts these compounds for further clinical studies [42]. Hence, this study clearly validates the potential of dibenzo[*b*,*f*][1,5]dioxocins as effective anti-GBM compounds by targeting the glutaminolysis pathway.

## 5. Conclusions

The novelty of the present work was focused on developing GLS-targeted dioxocins for treating GBM. Herein, details of the synthesis of several novel dioxocin derivatives along with their potential inhibitory activities on GBM cells through the inhibition of the glutaminolysis pathway have been discussed. All compounds were elucidated by analyzing pharmacokinetics data along with the structural interaction of the same compounds with GLS. Besides, methanodibenzo[*b*,*f*][1,5]dioxocins exhibited promising glutaminolysis pathway inhibitory activity, which was validated through the increased ROS production in GBM cells. This compound could occupy the major active site of GLS, which was confirmed by molecular docking. While the anti-GBM properties of many heterocyclic classes of compounds are well-established, to the best of our knowledge, this is the first investigation reporting the anti-GBM effects of a set of synthesized methanodibenzo[*b*,*f*][1,5]dioxocins. Further in vivo studies using methanodibenzo[*b*,*f*][1,5]dioxocins would help in identifying potential chemotherapeutic agents for treating glioblastoma.

## Figures and Tables

**Table 1:** Binding energy of dioxocins **1–7** docked with glutaminase.

Compounds	Binding Energy (kcal/mol)
**1**	−8.836
**2**	−9.816
**3**	−10.248
**4**	−9.576
**5**	−10.021
**6**	−9.820
**7**	−9.360

**Table 2:** In silico-predicted absorption, distribution, metabolism, excretion, and toxicity properties for dioxocins **1–7.** Abbreviation of descriptors are mentioned as follows: human gastrointestinal absorption (HIA), blood–brain barrier (BBB), central nervous system (CNS), and cytochromes P450 (CYP).

Properties	Descriptor	1	2	3	4	5	6	7
**Absorption**	Water solubility	Moderate	Moderate	Poor	Poor	Insoluble	Moderate	Poor
	HIA (% Absorbed)	97.448	96.131	94.899	97.544	88.634	99.653	93.225
	CaCO_2_ permeability (log Papp in 10 cm/s)	1.046	1.177	1.022	1.032	0.975	1.245	1.014
**Distribution**	P-glycoprotein substrate	Yes	No	Yes	Yes	Yes	No	Yes
	P-glycoprotein I inhibitor	Yes	Yes	Yes	Yes	Yes	Yes	Yes
	P-glycoprotein II inhibitor	Yes	Yes	Yes	Yes	Yes	Yes	Yes
	BBB permeability	Yes	No	No	No	No	No	No
	CNS permeability (log PS)	−1.751	−2.85	−1.155	−1.518	−0.912	−3.036	−1.309
**Metabolism**	CYP2D6 substrate	No	No	No	No	No	No	No
	CYP3A4 substrate	Yes	Yes	Yes	Yes	Yes	Yes	Yes
	CYP1A2 inhibitor	Yes	No	No	No	No	No	No
	CYP2C19 inhibitor	Yes	Yes	Yes	Yes	Yes	Yes	Yes
	CYP2C9 inhibitor	Yes	Yes	Yes	Yes	Yes	Yes	Yes
	CYP2D6 inhibitor	No	No	No	No	No	No	No
	CYP3A4 inhibitor	Yes	Yes	Yes	Yes	Yes	Yes	Yes
**Excretion**	Total Clearance (log mL/min/kg)	0.185	0.266	−0.393	0.113	−0.545	0.576	−0.197
	Renal OCT2 substrate	No	No	No	No	No	No	No
**Toxicity**	AMES toxicity	No	No	No	No	No	No	No
	Max. tolerated dose (human)(log mg/kg/day)	0.115	0.457	0.116	0.022	0.103	0.175	0.09
	Oral Rat Acute Toxicity (LD_50_, mol/kg)	2.516	2.977	2.805	2.598	2.733	2.838	2.707
	Hepatotoxicity	Yes	Yes	No	Yes	No	No	Yes
**Lipinski violations**		0	1	2	0	2	0	2

## Data Availability

The data are available from the corresponding authors upon request.

## Abbreviations

GBM, glioblastoma cancer cells; GLS, glutaminase; TMZ, temozolomide; DMEM, Dulbecco’s modified eagle medium; DMSO, dimethyl sulphoxide; ROS, reactive oxygen species; H2DCFDA, 2′,7′-dichlorodihydrofluoresceindiacetate.
